# New Methods of Clinical Pathology

**Published:** 1906-06

**Authors:** I. Walker Hall

**Affiliations:** Professor of Pathology, University College, Bristol; Pathologist to the Bristol Royal Infirmary


### NEW METHODS OF CLINICAL PATHOLOGY.

BY

I. Walker Hall, M.D.,

*Professor of Pathology, University College, Bristol; Pathologist to the Bristol Royal Infirmary.*

---

Coincident with the prosecution of investigations connected with questions of theoretical interest, considerable attention is being directed to the practical application of laboratory methods to clinical work. Some of the following tests and procedures are thus not only of interest from their theoretical significance, but promise to be widely employed by the clinician amongst his aids to diagnosis.

**Gastric Contents.**—A method of testing the gastric contents without the passage of the stomach tube has been devised by Sahli. It is based upon the supposition that the secreting capacity of the stomach can be better determined after a full meal than after the usual scanty so-called test meal. Immediately after the midday meal a methylene blue pill, enclosed in well-made catgut coverings (obtainable from G. Pohl, Schönbaum, Dantzig), is administered. The raw connective tissue, catgut, is only dissolved when the free hydrochloric acid is present in the gastric juice. The presence of blue pigment in the urine shows that secretion of acid by the stomach, digestion of the catgut, and absorption from the intestine are all being carried on; absence of it shows that food is passing too quickly from the stomach, or that there is insufficiency of the secretory or motor function; delay points to lesser degrees of insufficiency. It should be remembered that when the urine is alkaline the blue colour only becomes apparent after boiling with dilute acetic acid.

A number of workers agree that Gunsberg's is absolutely the best test for *free hydrochloric acid*, provided the solution is freshly mixed for each occasion.

For *quantitative estimations of acidity* the method advocated by Willcox is undoubtedly very correct. The total chlorides are determined by Volhard's method, the HCl. then driven off, and a second determination of the chlorides made. The latter result deducted from the former gives the *active* hydrochloric acid, and yields a most important factor for consideration in diagnosis.

From these figures it is possible frequently to determine the location of carcinoma to the cardiac or pyloric portions, and to indicate the presence of ulceration.

**Fæces.**—When examining the fæces for eggs of *intestinal parasites* the fæces should be made liquid in consistency by the addition of a small quantity of 10 per cent. formol solution. A number of smears should then be made on slides and fixed with 1 per cent. osmic acid vapour for two minutes. The films should then be mounted in glycerine or glycerine gelatine.

*Mucus* is best demonstrated by triturating the fæces with water in a mortar. The larger the masses of mucus the lower their origin in the intestine. Mucus from the small intestine is generally in small bile-stained particles.

*Starch* granules are easily recognised by the microscope. Their presence is always indicative of disease.

Schmidt tests for the presence of *bile* in the following manner:—A small quantity of fæces is diluted with water, and a concentrated solution of mercuric chloride is added, shaken, and the whole allowed to stand for twenty-four hours. Bile-stained material is coloured green, hydro-bilirubin stains red.

It is not advisable to examine for *fat* microscopically; quantitative determinations are absolutely necessary. Hecht proposes the following clinical method:—

1. Place 10 c.c. of fæces in a 300 c.c. flask.
2. Add a small piece of KOH and a little water.
3. After ten minutes add 100 c.c. 96 per cent. alcohol. Boil for twenty minutes.
4. Acidify with strong HCl, using 1 per cent. alcoholic alkali blue solution as indicator.
5. Filter; wash the filter with alcohol.

6. Evaporate to dryness. Take up the residue with ether.
7. Obtain an apparatus at a cost of 4s. 6d. from Haack, Vienna, and filter the ethereal solution into the lower part.
8. Allow the ether to evaporate. Fix the upper and lower parts of the apparatus together.
9. Add hot water (above 70° C.) until the melted fatty acids come to the top, and their percentage amount can be read off on the graduated table.

It is probable that this simple process will be widely employed for the diagnosis and treatment of nutritional disorders in children, and in diseases of the pancreas.

When *connective tissue* is present it points to gastric lesions, and may be recognised by staining for several hours in a dilute solution of Ehrlich's tri-acid, when it assumes a red colour. Acetic acid causes it to become transparent.

**Urine.**—Although an amount more than the daily normal average output of 1 gm. of *ammonia* is often present in the urine in disease, the trend of recent work is to indicate its value and limitations for diagnosis in inanition, diseases of the liver, and the onset of diabetic coma.

Its comparison with the total daily urea excretion no doubt furnishes a good idea of the extent of hepatic insufficiency.

A number of the newer methods consist of distillation in vacuo. Fürst describes a useful procedure. He adds methyl alcohol to the urine, in order to inhibit fermentation and to lower the boiling-point of the urine. The total daily urine is generally employed, but the ammonia output may be determined in the morning or in the evening urine. Immediately after the urine is passed and its quantity measured, 50 c.c. of methyl alcohol and 20 gm. NaCl are added to 50 c.c. of urine. This is distilled, together with 1 gm. of dry soda, into vessels containing $\frac{n}{10}$ $H_2SO_4$, a negative pressure of about 35–40 mm. Hg being maintained. The distillation is generally complete in about twenty minutes. The surplus acid is then determined by titration and the amount of ammonia calculated. No precautions are necessary for urines containing sugar or albumin.

Krokiewicz has proposed the following test for *bile* in the urine:—

Add equal amounts of a watery solution of sodium nitrite and of sulphanilic acid to a few drops of urine, and then pour in one or two drops of concentrated hydrochloric acid. The resultant solution is rubin red. When bile is present the colour becomes an amethyst violet. The colour is not removed by $CHCl_3$, ether, $CS_2$, and only slightly by amyl alcohol. A positive reaction indicates the presence of bili-rubin or other partially oxidised bile-stuffs.

It is not always an easy matter to definitely identify *albumose* in urine. When any such difficulty arises the following method of Fittipaldi may be used:—

Add 5 minims of a strong solution of trichloracetic acid to 5 c.c. of the urine, and remove any precipitate by filtration. To the filtrate add potassium mercuric iodide and filter. Suspend the precipitate in absolute alcohol, and centrifugalise. Pour off the alcohol and dissolve the sediment in 30 per cent. sodium hydrate solution. Add an ammoniacal solution of nickel sulphate (equal parts of ammonia and 5 per cent. solution of nickel sulphate). The presence of albumose is shown by an orange colouration.

Riegler has introduced a simple reaction for detecting the presence of *aceto-acetic acid* in the urine:—

To 1 c.c. of normal urine add 2 c.c. of 10 per cent. iodic acid solution, and 3 c.c. of $CHCl_3$. Shake. The $CHCl_3$ becomes violet. Add 10 c.c. of the urine to be investigated. When aceto-acetic acid is present the $CHCl_3$ is decolourised, the free iodine being taken up by the aceto-acetic acid.

*Acetone* occurs in all conditions of inanition, such as starvation, diabetes, fever, gastro-enteritis, nervous diseases, carcinoma, &c. Qualitative tests have limited its diagnostic value to cases of diabetes. Bluth has endeavoured to widen its application for diagnostic purposes by suggesting quantitative determinations, based upon the intensity of the colour reaction with sodium nitro-prusside, &c.

20 c.c. of urine are well shaken with 2 c.c. zinc chloride solution, and 1.5 c.c. of lead acetate added to the 15 c.c. of the clear filtrate. The mixture is again filtered, and 7.5 c.c. of the filtrate is mixed with an equal volume of sodium hydrate solution. The fluid is well shaken, and then thoroughly filtered. 10 c.c. are added to 1.5 c.c. of sodium nitro-prusside solution (1 to 9 aq. dest.). Using a control colour of 2 parts liq. ferri. sesquichlor. and 1 part of distilled water, the seconds occupied by the appearance of the red colour and its passage through orange to greenish yellow are counted. Twenty seconds are deducted for the influence of creatinin upon the reaction and the net time determined. Each second corresponds to 0.01 gm. acetone per litre of urine. When aceto-acetic acid is present, it is necessary to apply heat when it breaks up into acetone and $CO_2$. The amount of aceto-acetic acid may be ascertained by heating the urine with $\frac{n}{10}$ iodine solution and sodium hydrate solution, and then estimating the time of the decolourization.

**Tissues.**—The following method of Renaud is suitable for rapid examination of *nervous tissues*:—

1. Fix in 10 per cent. formol, or in equal parts of 10 per cent. formol and saturated $HgCl_2$ solution, twenty-four hours.
2. Wash for a few minutes in water.
3. Freeze and cut on freezing microtome.
4. Transfer sections to a solution of $\frac{1}{2000}$ to $\frac{1}{6000}$ osmic acid until the white substance becomes greyish brown: five to ten minutes.

For cellular structures overstain with aniline blue or phenic magenta; decolourize till the necessary details are obtained.

For axis cylinders and neuroglia, van Gieson's fluid is useful.

For myelin sheaths: overstain in 1 per cent. hæmatoxylin solution; treat with $\frac{1}{1000}$ permanganate of potash; decolourize with Pals solution; wash in water acidulated with nitric acid; dehydrate; mount in Canada balsam.

For degenerated myelin: After (3) transfer the sections to 5 per cent. chromic acid solution for one hour; then place in 1 per cent. osmic acid solution until they become yellowish-brown (about half an hour); place in 0.002 per cent. solution of tannic acid; wash in water acidulated with nitric acid; mount in lævulose syrup.

In order to stain the *spirochæta pallida* in sections, Levaditi fixes the tissue in formalin containing silver nitrate. It is then reduced in pyrogallic acid solution and washed in water. Sections are prepared and stained with undiluted Giemsa.

The spirochætæ are black, the cells blue, and the connective tissue yellow.

KÜHN (A.).—*München. med. Wchnschr.*, 1905, lii. 2412.

LETULLE (M.)—*Presse méd.*, 1905, p. 841.

SCHMIDT.—Quoted by Roux and Riva, *Gaz. d. Hôp.*, 1905, lxxviii. 807.

HECHT.—*München. med. Wchnschr.*, 1906, liii. 309.

FÜRST.—*Norsk Mag. f. Lægevidensk.*, 1905, lxvi. 1207.

KROKIEWICZ.—*München. med. Wchncshr*, 1906, liii. 496.

FITTIPALDI.—*Riforma med.*, 1905, No. 35.

RIEGLER.—*München. med. Wchnschr*, 1906, liii. 449.

BLUTH.—*Deutsche med. Wchnschr.*, 1906, No. 4.

RENAUD.—*Arch. gén. de Med.*, 1906.

LEVADITI.—*Ibid.*

